# A Novel Vaccine for Bovine Diarrhea Complex Utilizing Recombinant Enterotoxigenic *Escherichia coli* and *Salmonella* Expressing Surface-Displayed Chimeric Antigens from Enterohemorrhagic *Escherichia coli* O157:H7

**DOI:** 10.3390/vaccines13020124

**Published:** 2025-01-25

**Authors:** Hernán Ramírez, Daniel A. Vilte, Daniela Hozbor, Eugenia Zurita, Daniela Bottero, María C. Casabonne, Ángel A. Cataldi, Andrés Wigdorovitz, Mariano Larzábal

**Affiliations:** 1 Bioinnovo S.A., Hurlingham B1686, Argentina; hramirez@bioinnovo.com (H.R.);; 2 Instituto de Patobiología Veterinaria (IPVet) INTA-CONICET, Hurlingham B1686, Argentina; 3 Laboratorio VacSal, Instituto de Biotecnología y Biología Molecular, Facultad de Ciencias Exactas, Universidad Nacional de La Plata, CONICET, La Plata B1900, Argentina; 4 Instituto de Agrobiotecnología y Biología Molecular (IABIMO) INTA-CONICET, Hurlingham B1686, Argentina; 5 INCUINTA Instituto de Virología e Innovaciones Tecnológicas (IVIT) INTA-CONICET, Hurlingham B1686, Argentina

**Keywords:** bovine vaccine, immune response, EHEC O157:H7, EspB, intimin, ETEC, *Salmonella*, rotavirus, coronavirus

## Abstract

Background/Objectives: *Enterohemorrhagic Escherichia coli* (EHEC) O157:H7, a zoonotic pathogen primarily found in cattle, causes Hemolytic Uremic Syndrome (HUS) in humans, often through contaminated food. Its Type Three Secretion System (T3SS) facilitates gut colonization. In contrast, neonatal calf diarrhea (NCD) is mainly caused by pathogens like enterotoxigenic *Escherichia coli* (ETEC), *Salmonella* spp., Bovine Coronavirus (BCoV), and Bovine Rotavirus type A (BRoVA). This study engineered a chimeric protein combining EspB and Int280γ, two T3SS components, expressed in the membranes of *Salmonella* Dublin and ETEC. Methods: Immune responses in vaccinated mice and guinea pigs were assessed through ELISA assays. Results: Successful membrane anchorage and stability of the chimera were confirmed. Immune evaluations showed no enhancement from combining recombinant bacteria, indicating either bacterium suffices in a single formulation. Chimeric expression yielded immunogenicity equivalent to 10 µg of recombinant protein, with similar antibody titers. IgG1/IgG2a levels and Th1, Th2, and Th17 markers indicated a mixed immune response, providing broad humoral and cellular protection. Responses to BCoV, BRoVA, ETEC, and *Salmonella* antigens remained strong and did not interfere with chimera-specific responses, potentially boosting NCD vaccine efficacy. Conclusions: The chimera demonstrated robust immunogenicity, supporting its potential as a viable vaccine candidate against EHEC O157:H7. This approach could enhance NCD vaccine valency by offering broader protection against calf diarrhea while reducing HUS transmission risks to humans.

## 1. Introduction

Enterohemorrhagic *Escherichia coli* (EHEC) is a zoonotic pathogen that poses a significant public health concern. The most relevant serotype to human health is serotype O157:H7, known to cause diarrhea, hemorrhagic colitis and Hemolytic Uremic Syndrome (HUS) in humans [1]. HUS, a serious disease with global distribution, has a low incidence in industrialized countries such as the United States, Canada and Japan, with 1–3 cases per 100,000 children under the age of five [2]. In contrast, Argentina experiences endemic levels of HUS, particularly affecting young children [3]. Ruminants, especially cattle, are the primary reservoir of EHEC [4], with intermittent shedding of the bacteria through feces. Shedding is particularly high in young calves and around weaning the age [5].

Research indicates that vaccinating cattle against EHEC O157:H7 can effectively reduce colonization. A simulation based on data from the Health Protection Scotland’s enhanced surveillance system predicts that cattle vaccination could decrease EHEC O157:H7 shedding by approximately 50% and this decrease would ultimately reduce the cases in humans by 85% [6]. This underscores the potential of cattle vaccination as a strategy to lower food contamination and, consequently, reduce HUS cases.

Additionally, several EHEC O157:H7 virulence factors can elicit immune responses in cattle during natural or experimental infection. Research of experimental and natural infections has shown that calves produce serological responses to proteins encoded by the locus of enterocyte effacement (LEE) such as Intimin (bacterial adhesin) and the type 3 secretion system proteins EspA and EspB, and the bacterial receptor Tir [7].

The mouse model has been instrumental in evaluating antigenicity and immunogenicity in research on anti-EHEC candidate vaccines. For example, it has been used to test newborn mice’s protection against EHEC challenge [8], assess cytokine profile response after vaccination [9], compare immunization routes [10], explore novel EHEC vaccine formats [11], and test the efficacy of bivalent vaccines such as Brucella-EHEC [12], OMV-based vaccine formulations [13], chimeric antigens [14,15] and candidate conjugated vaccines [16].

On the other hand, Neonatal Calf Diarrhea (NCD) is a multifactorial disease in newborn cattle, primarily caused by pathogens such as enterotoxigenic *Escherichia coli* (ETEC F5+/K99), *Salmonella* spp., Bovine Coronavirus (BCoV) and Bovine Rotavirus type A (BRoVA) [17]. Current NCD vaccines available on the market generally contain inactivated ETEC and *Salmonella*. Based on promising findings from recombinant γ-intimin and EspB proteins of EHEC, we aimed to use these same proteins in the ETEC pathotype and *Salmonella* Dublin to stimulate an immune response against these microorganisms and potentially protect calves from NCD. Additionally, antibodies produced against these EHEC proteins, may reduce EHEC O157:H7 colonization in cattle, thereby helping to minimize food contamination.

Since existing bovine NCD vaccines also contain BCoV and BRoVA, this study also evaluated the combination of the recombinant bacteria with these viral particles to ensure no interference with the vaccine’s immunogenic properties. We assessed the immune response in mice and guinea pigs vaccinated with a recombinant chimeric protein, comprising the EspB and the C-terminal end of γ-intimin (Int280γ) antigens of EHEC O157:H7, anchored to the membrane of ETEC and *Salmonella* Dublin strains. This strategy aims to improve the efficacy of the NCD vaccine to protect calves against neonatal diarrhea while contributing positively to human health by reducing HUS transmission.

## 2. Materials and Methods

### 2.1. Production of EHEC Recombinant Proteins in ETEC and Salmonella Dublin

The chimeric sequence encoding EspB and Int280γ, fused via a linker, was synthesized by Genewiz (www.genewiz.com). The antigenic sequence also contained the coding sequences for Wza-Omporf1 in the N-terminal region, where Wza corresponds to the signal sequence of an enterobacterial lipoprotein and Omporf1 is an outer membrane protein of *Vibrio anguillarum* [18]. The chimera’s estimated molecular weight was 74 kDa. The nucleotide and aminoacidic sequence of the chimera is shown in Int280γ Supplementary Figure S1.

The chimeric construct included *Bam*HI and *Hin*dIII restriction sites at its ends, enabling its insertion into the pUC57 vector and subsequently into the pTrcHis2B vector (Invitrogen Corporation, Carlsbad, CA, USA). The pTrcHis2B vector with the corresponding ligated insert was transformed into *E. coli* DH5, and the successful ligation was then confirmed by PCR. The vector added a histidine and a c-Myc tag sequence to the chimera’s C-terminal end, facilitating detection and purification. This recombinant gene was designated BLI280.

Bacterial strains *E. coli* B41Arg (ETEC) and *Salmonella* enterica *serovar* Dublin 98/167 (*Salmonella* Dublin) were isolated in Argentina in the 1990 from cattle. Both strains were cultured in LB medium, and then transformed by electroporation with pTrcHis2B-BLI280.DublinDublin. Transformed bacteria were selected for ampicillin resistance conferred by the plasmid. Colonies of ETEC and *S.* Dublin transformed with the pTrcHis2B-BLI280 plasmid were inoculated and grown into 5 mL of MINCA broth with Vitox (for ETEC) and LB (for *S.* Dublin), respectively, each supplemented with 100 µg/mL ampicillin, at 37 °C with 200 rpm agitation. A 250 µL aliquot of each culture was inoculated into 25 mL of the respective media supplemented with ampicillin and grown with agitation until reaching a OD_600nm_ of 0.6–0.8. At this point, protein expression was induced with 1 mM IPTG (isopropyl-β-D-1-thiogalactopyranoside) for 4 h with 200 rpm agitation at 37 °C. The cultures were then centrifuged (3000× *g*, 10 min, 4 °C), and the resulting pellets were retained for vaccine formulation after inactivation. This vaccine formulation was used to inoculate mice in groups 5, 6, 7 and 8, as well as the guinea pig vaccination group. Recombinant His-tagged Int280γ, EspB and Chimeric proteins were prepared as previously described [19]. Briefly, the gene fragment (843 bp) encoding the 280 carboxyl-terminal amino acids of γ -Intimin and EspB (936 pb) gene were amplified by PCR from a bovine EHEC O157:H7 isolation. Moreover, the chimera sequence (2079 bp) was also amplified by PCR from the synthesized sequence performed in Genewiz. The amplicon was cloned into the His-tag expression vector pRSET-A (Invitrogen, Carlsbad, CA, USA). The resulting constructs were subsequently transformed into chemically competent *E. coli* BL21 (D3)/pLysS cells.

Protein expression was induced using 1 mM IPTG. Bacterial lysates were prepared, and His-tagged proteins were purified by affinity chromatography using nickel-agarose columns (ProBond Nickel-Chelating Resin, Invitrogen). The purified proteins were eluted under denaturing conditions and subsequently dialyzed in PBS (pH 7.4).

This vaccine formulation, containing the recombinant proteins Int280γ and EspB, was used to inoculate mice in group 2, while groups 3 and 4 were inoculated with the chimera recombinant protein. These recombinant proteins were also used to coat ELISA plates.

### 2.2. Inactivation of Recombinant ETEC and Salmonella Dublin

Recombinant bacteria were cultured at 37 °C in media optimized for protein expression, and then centrifuged at 16,400× *g* for 10 min. Pellets were resuspended in formalin (40% stabilized with methanol) in PBS pH 7.4 (137 mM NaCl, 2.7 mM KCl, 10 mM Na_2_HPO_4_, 1.8 mM KH_2_PO_4_). Inactivation was performed by shaking the bacterial suspensions at 150 rpm for 1 h at room temperature, followed by incubation at 4 °C. The suspensions were inactivated using varying concentrations of formalin (0.2% and 0.3% *v*/*v*), incubation times (8, 24 and 72 h), and temperatures (4 and 37 °C). Inactivation was confirmed by plating samples of the treated bacterial suspension on Trypticase Soy agar and incubating at 37 °C for 7 days.

### 2.3. Cellular Localization of the Chimera Protein Detection

Chimera expression and localization on the outer membrane was analyzed as previously described [18]. Bacterial-induced cultures were centrifuged at 10,000× *g* for 2 min, washed three times with PBS, and resuspended in 1.5 mL of Tris-HCl-NaCl (50 mM, pH 8.0, 0.3% NaCl) buffer.

The cell suspension was sonicated on ice for 5 min, using a 10 s on/off pulse cycle and 20–50% amplitude. The supernatant was centrifuged at 10,000× *g* for 1 h at 4 °C to remove the unbroken cells and debris and isolate the total membrane fraction. This new supernatant was considered the soluble cytoplasmic/periplasmic fraction.

Outer membrane fractionation was performed by resuspending the pellet in 0.4 mL of HEPES buffer (10 mM [pH 7.4], containing 1% sodium lauryl sarcosine) to solubilize the inner membrane. After a 30 min incubation at room temperature, the mixture was ultracentrifuged at 20,000× *g* for 1 h at 4 °C to isolate the outer membrane fraction. Samples were stored for further analysis.

### 2.4. Western Blot Assay

Samples from inactivation and fractionation procedures were analyzed by Western blot. Proteins were separated by SDS-PAGE using a 12% polyacrylamide gel under reducing conditions and transferred onto a nitrocellulose membrane (Merck KGaA, Darmstadt, Germany). The membranes were blocked with 3% nonfat dry milk prepared in PBS (pH 7.4) for 1 h at room temperature. with agitation, washed with PBS-T, and incubated with serum samples for 1 h. After washing, membranes were incubated with HRP-conjugated rabbit anti-bovine IgG (Bethyl Laboratories, Montgomery, TX, USA) diluted 1:5000 in PBS-T for 1 h. Signals were developed using 3,3′-Diaminobenzidine (DAB) (Pierce, Rockford, IL, USA).

Western blot was performed on serum samples collected before and after immunization to confirm antibody specificity. Serum from a bovine inoculated with recombinant EspB and Int280γ antigens (1:500 dilution) served as a positive control.

### 2.5. Strains and Production of Coronavirus and Rotavirus

Bovine Rotavirus UK (BRV UK) and Bovine Coronavirus (BCoV) Mebus strains were cultured in D-MEM (Gibco, Thermo Fisher, Waltham, MA, USA) supplemented with 2 µg/mL trypsin and antibiotics. BRV UK was propagated in MA-104 monkey kidney cells, and BCoV in MDBK bovine kidney cells, incubated at 37 °C with 5% CO_2_. Cytopathic effects were monitored at 48–72 h post-infection. Viral particles were released by freeze–thaw cycles, clarified by centrifugation at 3000× *g* for 20 min, and supernatants stored at −80 °C. Inactivation was performed using 0.5% formalin for 48 h at 4 °C.

### 2.6. Fluorescent Focus Reduction Assay

The virus neutralization (VN) assay was performed as previously described [20]. Guinea pig sera were heat-inactivated at 56 °C for 30 min. Serial four-fold dilutions (1:4 to 1:1024) were mixed with equal volumes of Group A Rotavirus (100 FFU/100 µL) and incubated at 37 °C for 1 h. MA-104 cells (200,000 ± 50,000 cells) were added and incubated for 3 days at 37 °C. Plates were fixed with 70% acetone, and detection was performed using fluorescein isothiocyanate-labeled anti-RV polyclonal antiserum.

Tests were valid if virus titration resulted in 100 TCID50 (±50–200 TCID50). Positive controls yielded expected titers (±1 SD), and negative serum showed no neutralization. VN Ab titers were calculated by the Reed and Muench method [21]. Negative samples were assigned an arbitrary value of 0.30 for calculations.

### 2.7. Animal Models and Immunization

Male BALB/c mice (3 months old) and female Hartley guinea pigs (4 months old) were used as animal models. Mice were divided into eight groups, with seven groups containing five vaccinated mice each, and one control group with four animals. Each animal received two subcutaneous vaccine doses in the neck at 21-day intervals, using ISA 50 as the adjuvant in a 50:50 aqueous phase ratio. Groups 1–8 were vaccinated according to Table 1A. Serum samples were collected on days 1 and 21 post-vaccination. Additional samples were taken on days 39 and 70 for guinea pigs and day 39 for mice.

The guinea pig cohort consisted of ten animals divided into two groups of five. Each animal received two vaccine doses, administered subcutaneously in the neck area at 21-day intervals, using ISA 50 as the adjuvant in a 50%:50% ratio with respect to the aqueous phase. The groups were numbered as 1 and 2 and vaccinated according to the specifications in Table 1B.

Animals were housed in ventilated cages under standardized conditions, including controlled light cycles, humidity, and temperature. Food and water were provided *ad libitum*. The animal experiments were authorized by the Institutional Committee for the Care and Use of Experimental Animals CICUAE INTA CICVyA approved the study (CICUAE 09/2023).

### 2.8. IgG Specific Antibody ELISA

Specific IgG antibody titers against the chimera, Int280γ, and EspB were measured in sera samples from mice and guinea pigs using an indirect ELISA. Briefly, 96-well ELISA plates (Ivema, ES08 Buenos Aires, Argentina) were coated overnight at 4 °C with 500 ng/well of each protein in carbonate/bicarbonate buffer (pH 9.6). The plates were washed with PBS containing 0.05% Tween 20 (PBS-T, pH 7.4) and blocked with 3% nonfat dry milk for 1 h at 37 °C to prevent non-specific binding. Four-fold serial dilutions of serum samples in 3% nonfat dry milk were added (100 µL/well), and the plates were incubated for 1 h 37 °C. Each plate included a blank control with 3% nonfat dry milk alone, a known positive sample, and a negative sample. After washing with PBS-T, the plates were incubated for 1 h with 100 µL of goat anti-mouse IgG or goat anti-guinea pig conjugated with horseradish peroxidase (Bethyl Laboratories, Montgomery, TX, USA), both at a dilution of 1:4000 in 3% nonfat dry milk. The plates were washed four times with PBS-T followed by the addition of ABTS substrate [2,2′-azino-bis(3-ethylbenzthiazoline-6-sulphonic acid)] (Amresco, Solon, OH, USA), which was prepared in citrate-phosphate buffer (pH 4.2) containing 0.01% H_2_O_2_ and added at 100 µL per well. Reactions were stopped after 10 min by adding 100 µL per well of 5% SDS. Absorbance was measured at 405 nm (OD_405_) using a BioTek ELx808 microplate reader (BioTek Instruments, Winooski, VT, USA)

Antibody titers were expressed as the logarithm of the reciprocal of the endpoint dilution that produced an OD_405_ exceeding the cut-off value. The cut-off was defined as the mean optical density of day 0 samples plus two standard deviations.

### 2.9. Specific Antibody BCoV and BRoVA ELISA

Antibody titers against BCoV and BRoVA in both animal models were determined using a double-sandwich ELISA [22]. For the viral ELISA assays, hyperimmune sera from cattle immunized with commercial doses provided by BIOINNOVO were used to coat the plates Briefly, 96-well plates were coated with hyperimmune anti-BCoV serum in carbonate-bicarbonate buffer (pH 9.6) and incubated for 18 h at 4–8 °C. Plates were blocked with 10% nonfat milk in PBS-T. Clarified supernatants from HRT-18 or MA-104 cell cultures infected with a standardized titer of coronavirus were added. Supernatants from uninfected cells served as controls. Two-fold serial dilutions of the samples, along with positive and negative controls, were then added to the wells.

Two-fold serial dilutions of the samples and their respective positive and negative controls were added to the wells. Finally, commercial polyclonal anti-mouse or anti-guinea pig antibodies conjugated to peroxidase were added, depending on the species. The reaction was developed using H_2_O_2_ and ABTS, and then stopped with SDS, and the absorbance was measured at 405 nm (Multiskan FC, Waltham, Massachusetts, US,).

### 2.10. Antigen- Specific IL-17, IFN-γ and IL-5 Production by Spleen Cells

Spleens from untreated and immunized mice were processed through a 40 µm cell strainer to obtain single-cell suspensions. Spleen cells were plated in 48-well culture plates at a final volume of 500 µL per well in RPMI 1640 medium supplemented with 10% fetal bovine serum, 100 IU/mL penicillin, and 100 µg/mL streptomycin. Cell samples were stimulated with either 2 µg/mL antigen or medium alone as a control. After 72 h of incubation at 37 °C in a 5% CO_2_ atmosphere, concentrations of IFN-γ, IL-5, and IL-17 in the supernatants were measured using ELISA (BD Biosciences, San Diego, CA, USA) according to the manufacturer’s instructions.

### 2.11. Statistical Analysis

The normality of the immune response assay data was assessed using the Shapiro–Wilk test, which indicated a normal distribution (*p* > 0.05). The data were statistically analyzed using one-way or two-way analysis of variance (ANOVA), followed by Bonferroni’s post hoc test for multiple comparisons, performed with GraphPad Prism^®^ version 5.0 software. Differences were considered statistically significant at *p* < 0.05.

## 3. Results

### 3.1. Design, Cloning and Introduction into Carrier Bacteria of Chimera Protein

The chimeric protein was designed as a composite sequence from N- to C-terminus, structured as Wza-Omporf-EspB-linker-Int280γ. The gene encoding this chimera, designated BLI280, was synthesized de novo and initially cloned into a cloning vector. Following excision, the gene was inserted into the expression vector pTrcHis2B, generating pTrcHis2B-BLI280. This construct was introduced into *Salmonella enterica* serovar Dublin and enterotoxigenic *Escherichia coli* (ETEC) via electroporation.

### 3.2. Membrane-Anchoring of Chimeric Protein to ETEC and Salmonella Dublin

**Figure 1:**
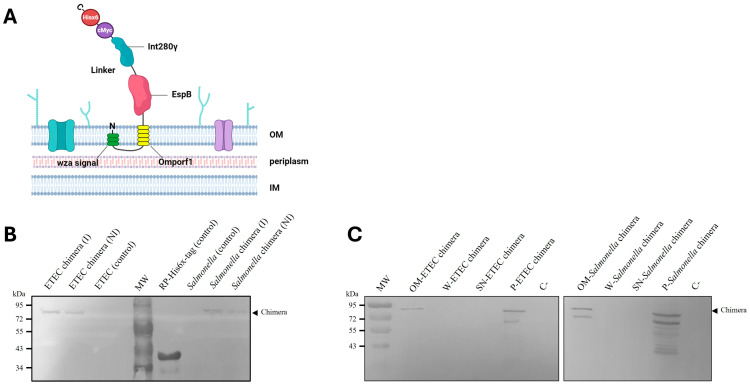
(**A**) Schematic representation of the chimeric protein. The chimera comprises a Wza-Omporf1 anchor sequence that inserts itself into the outer membrane (OM) at the N-terminal end and orients the rest of the EspB-linker-Int280 antigenic fusion to the extracellular milieu. The C-terminal region ends with the epitopes for c-Myc and His6x-tags. The approximate molecular weight of the chimera is 74 kDa. Detection of the recombinant chimeric protein expression and outer membrane localization in recombinant ETEC and *Salmonella*. (**B**) ETEC and *Salmonella* transformed with pTrcHis2B-BLI280 were induced (I), or not (non-induced: NI), with IPTG for recombinant chimera expression. The samples were separated by SDS-PAGE. Wild-type ETEC and *Salmonella* strains were used as controls not expressing the chimera and recombinant Int280ϒ-Hisx6 (RP) purified protein was used as primary antibody control. (**C**) ETEC and *Salmonella* transformed with pTrcHis2B-BLI280 were induced with IPTG to express the recombinant chimera. Samples of different fractions from the membrane purification process of ETEC and *Salmonella* were separated by SDS-PAGE. References: outer membrane (OM), pellet washings (W), pellet supernatant (SN), total bacterial pellet (P) and total bacterial culture supernatant (C). Chimera detection was performed using a mouse-specific anti Hisx6-tag primary antibody and an alkaline phosphatase-conjugated anti-mouse as a secondary antibody, in both Western blot assays.

The anchorage of the chimeric protein to the bacterial membranes (Figure 1A) in ETEC and *Salmonella* Dublin transformed with pTrcHis2B-BLI280 was assessed using Western blot analysis of cytoplasmic and membrane fractions obtained by ultracentrifugation. Both induced and non-induced bacteria were analyzed. Detection of the chimera in Western blots was performed using an anti-histidine tag (Hisx6) antibody and bovine serum specific to the EspB and Int280γ antigens, as described in Section 2.

The presence of the chimeric protein was confirmed in both ETEC and *Salmonella* samples, regardless of IPTG induction (Figure 1B,C). Furthermore, the chimeric protein was localized exclusively to the outer membrane fraction in both bacterial species, as evidenced by the absence of the protein in pellet washings and pellet supernatants (Figure 1B,C). Additional analyses using bovine serum specific to EspB and Int280γ (Supplementary Figure S2) further validated the outer membrane localization of the chimera. These results confirm that the chimeric protein is successfully anchored to the outer bacterial membrane.

### 3.3. Inactivation of Recombinant ETEC and Salmonella Dublin and Antigenic Preservation in Vaccine Formulation

**Figure 2:**
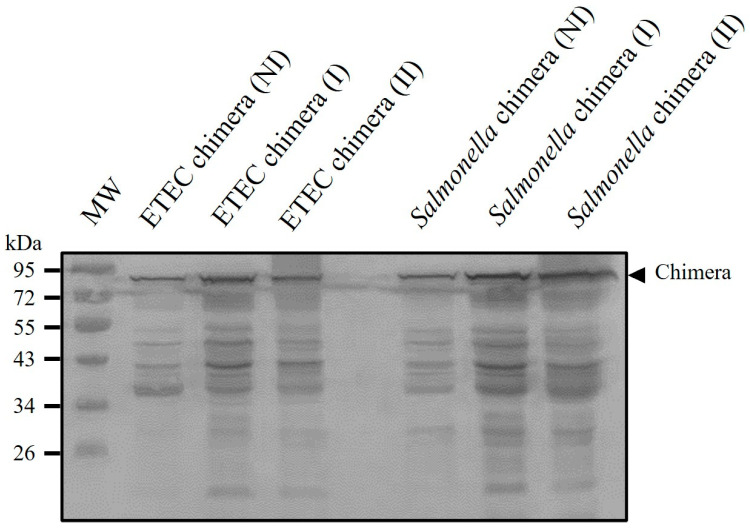
Detection of recombinant chimeric protein in inactivated recombinant ETEC and *Salmonella*. Induced recombinant ETEC and *Salmonella* were inactivated with 0.2% formalin incubated for 72 h at 4 °C. The samples were separated by SDS-PAGE. Chimera detection was performed using a mouse-specific anti Hisx6-tag primary antibody and an alkaline phosphatase-conjugated anti-mouse as a secondary antibody. The abbreviations correspond to uninduced bacteria (NI), induced bacteria (I) and induced and inactivated bacteria (II).

Inactivation assays conducted on induced suspensions of recombinant chimeric ETEC (see Section 2 and Supplementary Figure S3) showed that the optimal inactivation condition was 0.2% *v*/*v* formalin for 96 h at 4 °C. This treatment caused a slight decrease in chimeric protein concentrations, as observed through Western blot analysis (Figure 2). The preservation and integrity of the antigen in recombinant ETEC and *Salmonella Dublin* were also assessed after storage at 4 °C for 2 to 7 months, with results confirming stability via Western blot (Supplementary Figure S4).

### 3.4. Immune Response of Mice and Guinea Pigs

**Figure 3:**
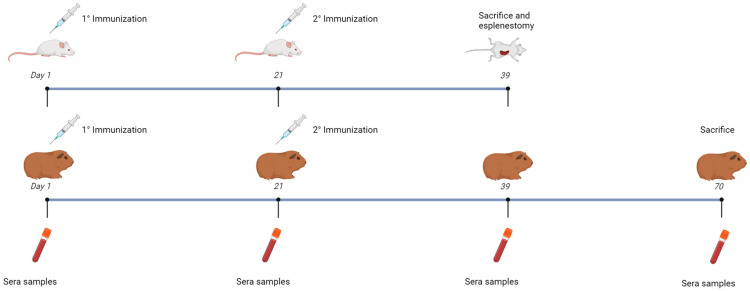
Scheme of immunization assays of mice and guinea pigs. Groups of mice and guinea pigs were immunized with two doses of vaccine preparations in an interval of 21 days via subcutaneous injection. Sera samples were collected from both animal models at days 1, 21 and 39. On day 39, the mice were sacrificed, and splenectomy was performed. An additional sera sample collection was performed for the guinea pigs on the 70th day before performing euthanasia of the animals. The mice were divided into seven groups of five animals each; one group of four animals was used as a control. The different groups were numbered from 1 to 8 and inoculated with the following antigens combinations: Group 1: PBS Control, Group 2: 1 µg of EspB and 1 µg of Int280ϒ, Group 3: 2 µg of the chimera, Group 4: 10 µg of the chimera, Group 5: ETEC expressing the chimera, Group 6: *Salmonella* expressing the chimera, Group 7: Both recombinant bacteria expressing the chimera and Group 8: Both recombinant bacteria expressing the chimera plus BCoV and BRoVA viral particles. The guinea pigs were divided into two groups of five animals each. One group was inoculated with a vaccine containing both recombinant bacteria expressing the chimera plus BCoV and BRoVA viral particles. The other group was vaccinated with PBS (Control).

The immunogenicity and efficacy of the vaccine strategy targeting the bovine diarrhea complex, incorporating the recombinant EHEC O157:H7 antigen, were evaluated through immune response assays in mice and guinea pigs (Figure 3).

**Figure 4:**
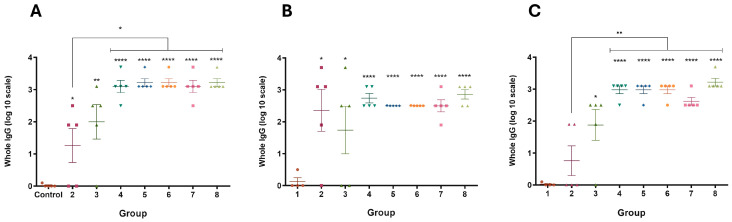
Specific IgG responses at 39 dpv in inoculated mice. ELISA plates were coated with purified the recombinant chimera (**A**), EspB (**B**) and Int280ϒ (**C**), respectively. Specific antibodies response to the chimera, Int280ϒ and EspB were measured for each group using indirect ELISA and sera samples (39 dpv) from mice. Group 1: 150 µL of PBS (Control), Group 2: 1 µg of EspB and 1 µg of Int280γ, Group 3: 2 µg of the chimera, Group 4: 10 µg of the chimera, Group 5: 1.10^8^ inactivated CFU of ETEC B41Arg expressing the chimera, Group 6: 1.10^8^ inactivated CFU of *Salmonella* Dublin expressing the chimera, Group 7: 1.10^8^ inactivated CFU of ETEC B41Arg and *Salmonella* Dublin, both expressing the chimera and Group 8: 1.10^8^ inactivated CFU of ETEC B41Arg and *Salmonella* Dublin, both expressing the chimera, plus 1.10^7^ FFU BRoVA UK and BCoVB Mebus. Goat anti-mouse IgG conjugated with horseradish peroxidase was used as a secondary antibody. ABTS was used as substrate and the reaction was measured at OD_405_. The antibody titer was expressed as the reciprocal of the end-point dilution resulting in an OD_405_ above the cut-off value. The cut-off value was calculated as the average plus two times the standard deviation of the optical densities of the samples measured on day 0. Statistical analysis by ANOVA, *p* < 0.05 (*), *p* < 0.01 (**) and *p* < 0.0001 (****).

In mice, at 39 days post-vaccination (dpv), groups receiving individual antigens (Group 2) or the chimeric fusion antigen (Groups 3 and 4) exhibited higher immune responses compared to the control group (Group 1) (Figure 4A). Comparable molecular amounts of the individual antigen (Group 2) and the chimera (Group 3) produced similar immune responses. The immune response from 10 µg of recombinant chimera (Group 4) was equivalent to that in groups using ETEC and/or *Salmonella Dublin* expressing the recombinant chimera in their membranes (Groups 5 to 8). Adding 10⁷ focus-forming units (FFU) of BCoV and BRoVA into the vaccine mixture did not impair the immune response to the chimeric protein (Group 8 vs. Group 7). Both soluble and membrane-expressed chimera vaccinations induced specific immune responses against Int280γ and EspB (Figure 4B,C). No significant differences were found in the immune response between single-dose and booster vaccinations in Groups 4 to 8 (Supplementary Figure S5), suggesting a single dose might suffice.

**Figure 5:**
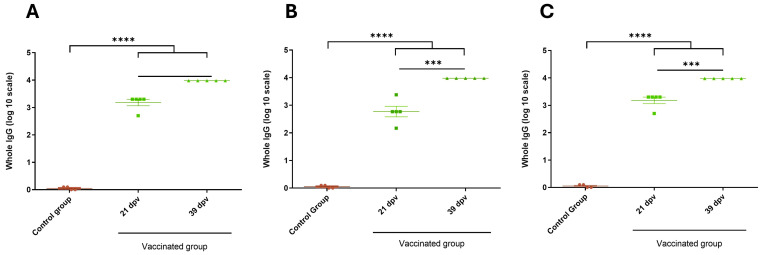
Specific IgG responses in inoculated guinea pigs. ELISA plates were coated with the purified recombinant chimera (**A**), EspB (**B**) and Int280ϒ (**C**), respectively. Specific antibodies response to the chimera, Int280ϒ and EspB were measured for each group using indirect ELISA and sera samples from guinea pigs. Goat anti-guinea pig IgG conjugated with horseradish peroxidase was used as a secondary antibody. ABTS was used as substrate and the reaction was measured at OD_405_. The antibody titer was expressed as the reciprocal of the end-point dilution resulting in an OD_405_ above the cut-off value. The cut-off value was calculated as the average plus two times the standard deviation of the optical densities of the samples measured on day 0. Statistical analysis by ANOVA *p* < 0.0002 (***) and *p* < 0.0001 (****).

In guinea pigs, the complete vaccination strategy (Group 8) elicited a strong immune response against the chimera, Int280γ, and EspB, comparable to results observed in mice (Figure 5). The immune responses against Int280γ and EspB showed no significant differences, indicating similar immunogenicity for both antigens (Figure 5B,C).

### 3.5. Evaluation of Immune Response Against Chimera-Carrying Bacteria

**Figure 6:**
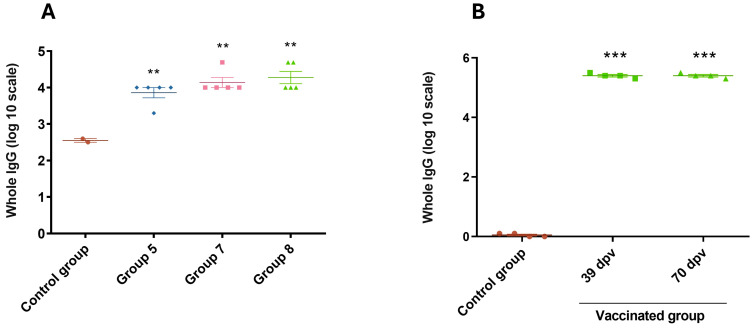
IgG response against fimbria in sera of mice and guinea pigs inoculated with ETEC. ELISA plates were coated with purified fimbriae. Antibody response to fimbriae was measured for each group using indirect ELISA and sera samples from mice (**A**) and guinea pigs (**B**). Goat anti-guinea pig IgG conjugated with horseradish peroxidase was used as a secondary antibody. ABTS was used as a substrate, and the reaction was measured at OD_405_. The antibody titer was expressed as the reciprocal of the end-point dilution resulting in an OD_405_ above the cut-off value. The cut-off value was calculated as the average plus two times the standard deviation of the optical densities of the samples measured on day 0. Statistical analysis by ANOVA, *p* < 0.01 (**) and *p* < 0.001 (***).

**Figure 7:**
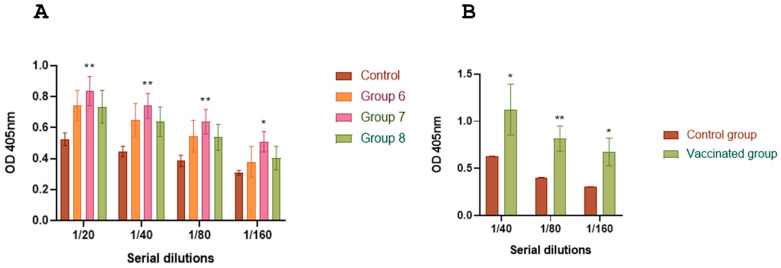
IgG response against LPS in sera of inoculated mice and guinea pigs with *Salmonella*. ELISA plates were coated with purified LPS. Antibodies response to LPS were measured for each group using indirect ELISA and sera samples from of mice (**A**) and guinea pigs (**B**). Goat anti-guinea pig IgG conjugated with horseradish peroxidase was used as a secondary antibody. ABTS was used as a substrate and the reaction was measured at OD_405_. The antibody titer was expressed as the reciprocal of the end-point dilution resulting in an OD_405_ above the cut-off value. The cut-off value was calculated as the average plus two times the standard deviation of the optical densities of the samples measured on day 0. Statistical analysis by ANOVA, *p* < 0.05 (*) and *p* < 0.01 (**).

To integrate the chimera antigen in an existing vaccine formulation, such as the one for bovine neonatal diarrhea, we evaluated the immune responses induced by chimera-carrying bacteria and viral particles in mice and guinea pigs. ELISA analysis of total specific IgG levels against ETEC fimbriae (Groups 5, 7, and 8) and *Salmonella* lipopolysaccharide (LPS) (Groups 6, 7, and 8) in mice confirmed immune responses to both fimbriae and LPS (Figure 6A and Figure 7A). Similar results were observed in guinea pigs inoculated with the complete vaccine formulation (Figure 6B and Figure 7B). In guinea pigs, the basal fimbriae response was notably lower than that observed in control groups inoculated with chimera-carrying bacteria (Figure 6A,B).

### 3.6. Antibody Titers Against BRoVA UK and BCoV Mebus

**Figure 8:**
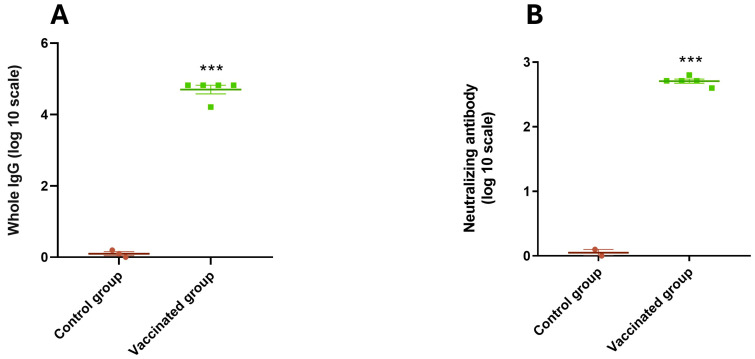
IgG response against BCoV and neutralizing antibodies against BRoVA in sera of vaccinated guinea pigs. (**A**) ELISA plates were coated with hyperimmune anti-BCoV serum. Clarified supernatants from HRT-18 cultures infected with standardized titer of coronavirus or supernatants from uninfected cells (control) was added into the corresponding wells. Commercial polyclonal anti-mouse or anti-guinea pig antibodies conjugated to peroxidase were added as appropriate. The plates were read using an ELISA reader at 405 nm. (**B**) Mixtures of serial dilutions of guinea pig serum were incubated with equal amounts of BRoVA. The mixture was incubated with a cell suspension to determine neutralization. The test was developed using a fluorescein isothiocyanate-labeled anti-RV polyclonal antiserum derived from a colostrum-deprived calf by hyperimmunization. Statistical analysis by ANOVA, and *p* < 0.001 (***).

Guinea pigs receiving the complete vaccine formulation exhibited significantly higher antibody titers against BCoV Mebus compared to the control group (Figure 8A). Virus neutralization assays using BRoVA UK demonstrated that vaccinated guinea pigs had higher neutralizing antibody titers (512) than controls (Figure 8B).

### 3.7. Antigen-Specific Immune Response Profiles Across Vaccination Groups

**Figure 9:**
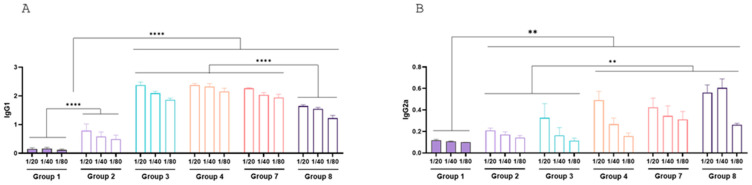
Mice antibody titers induced by recombinant chimera. Total titers of IgG1 (**A**) and IgG2a (**B**). Groups are formed as in Figure 4 Group 1: 150 µL of PBS (Control), Group 2: 1 µg of EspB and 1 µg of Int280γ, Group 3: 2 µg of the chimera, Group 4: 10 µg of the chimera, Group 7: 1.10^8^ inactivated CFU of ETEC B41Arg and *Salmonella* Dublin, both expressing the chimera and Group 8: 1.10^8^ inactivated CFU of ETEC B41Arg and *Salmonella* Dublin, both expressing the chimera, plus 1.10^7^ FFU BRoVA UK and BCoVB Mebus.. Isotypes were determined in serum dilutions from various vaccinated groups using an indirect ELISA with a purified recombinant chimera as the antigen. Titers are expressed as geometric mean of each group (n = 5). Statistical analysis was performed by Bonferroni test, *p* < 0.01 (**) and *p* < 0.0001 (****).

Mice vaccinated with 2 µg of chimera (Group 3), 10 µg of chimera (Group 4), or 10⁸ inactivated CFU of chimera-expressing ETEC and *Salmonella Dublin* (Group 7) exhibited the highest IgG1 levels (Figure 9A). Group 8, receiving the chimera with BCoV and BRoVA, showed slightly lower IgG1 levels than other groups (*p* < 0.0001).

For IgG2a, Groups 4, 7, and 8 demonstrated the highest levels, while Groups 2 and 3 also displayed significantly elevated levels compared to controls (Figure 9B).

**Figure 10:**
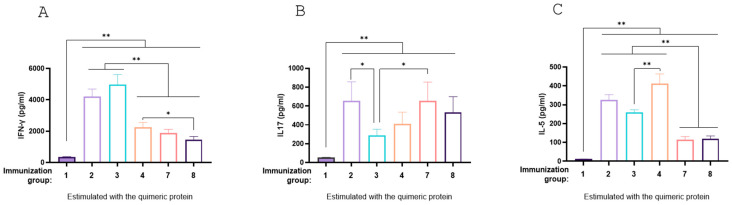
Cytokine production by splenocytes from immunized mice. BALB/c mice were not immunized (control) or immunized with different vaccine formulations. Eighteen days after the last immunization, mice were sacrificed, and spleen cells were stimulated with purified recombinant chimera. After 72 h of culture, the concentrations of IFN-γ (**A**), IL-17A (**B**) and IL-5 (**C**) were determined in the culture supernatant by ELISA. The results are expressed as mean values (±standard error) of three experiments with 5 mice per group. Significant differences were analyzed for each cytokine between different vaccines for each stimulus, *p* < 0.05 (*) and *p* < 0.01 (**).

Splenocyte stimulation assays revealed that all treatments (Groups 2, 3, 4, 7, and 8) elicited a mixed Th1, Th2, and Th17 profile. Groups 7 and 8 had reduced IFN-γ and IL-5 levels but exhibited elevated IL-17 levels, significantly higher for Group 7 compared to Group 3 (*p* < 0.05, Figure 10A–C). These findings suggest robust antigen-specific immune responses induced by both recombinant and carrier-expressed formulations.

## 4. Discussion

Proteins from T3SS have been explored as potential components for rational design of vaccines aimed at reducing fecal shedding of EHEC O157:H7 in cattle [23,24,25]. Previous studies have demonstrated that intramuscular vaccination with recombinant EspB and Int280γ antigens elicits a specific humoral immune response in cattle serum [8] and induces a specific mucosal secretory immune response through oral vaccination in mice [26]. Additionally, these specific antibodies can inhibit adhesion to epithelial cell lines, reduce T3SS-dependent red blood cell lysis, and diminish colonization and shedding in both bovines [19] and mice orally challenged with EHEC O157:H7 [26]. These findings highlight the potential of an intramuscular vaccine containing these antigens, though optimization of the formulation is required to enhance protection and ensure commercial feasibility for cattle vaccination.

In this study, we designed a membrane-anchoring chimeric protein combining the EspB and Int280γ antigens and evaluated its expression in two pathogens responsible for NCD: *Salmonella* Dublin and ETEC. The chimera was successfully expressed in the bacterial membrane, confirming correct translation of the coding sequence for Wza-Omporf1 and the His(6X)-cMyc tag at the N- and C-terminal regions, respectively. Expression analysis revealed basal chimera expression in non-induced bacteria, while induced strains exhibited consistently higher expression levels. The chimera also demonstrated stability under inactivation and storage conditions. Chimeric proteins, also known as fusion proteins, are increasingly utilized as vaccinal antigens in experimental and preclinical vaccine studies, as well as in licensed vaccines [27,28,29,30]. These proteins can encode fused antigenic domains derived either from two or more different pathogens or from distinct proteins of the same pathogen, as observed in our study [31]. The display of antigens on the surface of live attenuated or inactivated bacterial vaccines facilitates their recognition by the immune system, likely through the accelerated detection of pathogen-associated molecular patterns (PAMPs) by antigen-presenting cells, such as dendritic cells and macrophages [32,33,34]. Surface display not only represents an attractive strategy for presenting heterologous antigens to the host immune system but also enhances the immunogenicity of these antigens. By maintaining their native or near-native conformation, surface-displayed antigens improve recognition by B cells, promoting the production of specific antibodies, including neutralizing antibodies. Furthermore, these systems enable multivalency, presenting multiple copies of the antigen simultaneously, which significantly amplifies B cell activation and strengthens humoral immune responses [35,36,37].

The IgG responses to fimbriae and LPS observed in serum samples indicate the preservation of bacterial structures, an essential factor for future scale-up and licensing efforts. Immune responses from sera of both experimental models inoculated with individual or combined recombinant bacteria exhibited high titers of chimera-specific antibodies. These results support proper expression, anchorage, and membrane exposure of the chimera, as well as effective preservation of the vaccine antigen even after inactivation and storage. In immunization assays, no significant differences were observed between the use of individual recombinant bacteria and their combination, suggesting saturation of the immune response. This phenomenon of immune response saturation occurs when the immune system reaches its maximum capacity to respond to an antigen, regardless of additional doses. This is due to the finite availability of antigen-specific lymphocytes, receptor occupancy, and the limited capacity of antigen-presenting cells. Recognizing this phenomenon is crucial in vaccine development, as it helps optimize immunogenicity without unnecessarily overloading the system with additional antigens. In the absence of a detectable synergistic effect, a vaccine formulation containing either recombinant ETEC or *Salmonella* expressing the chimera alone would likely be sufficient. However, future studies should assess the neutralizing activity generated by both bacterial carriers against EHEC O157:H7 colonization in cattle.

Regarding chimera expression in the bacterial membrane, 1×10⁸ CFU of bacteria expressing the chimera were immunologically equivalent to 10 µg of the recombinant chimeric antigen. Immune responses at 21 and 39 days post-vaccination (dpv) were comparable regardless of dose in both models, suggesting that a single vaccination may suffice in a potential vaccination strategy. Furthermore, individual evaluations of the EspB and Int280γ antigens elicited comparable immune responses, underscoring their importance in chimera construction and robust immunogenic properties.

Although no specific studies have been conducted to evaluate the lack of interference between these antigens within a single vaccine, the existence and widespread use of multivalent vaccines in veterinary practice (e.g., Bovisan Diar, Rotavec^®^ Corona, Fencovis, Block Neonatal) support the notion that combining these antigens is effective and does not result in significant interactions that could compromise the desired immune response.

Immunological analyses of responses to BCoV and BRoVA demonstrated that these viral particles induced strong, antigen-specific immune responses without impairing the response to the chimera. Similarly, LPS and fimbriae antigens elicited robust immune responses in both models evaluated. However, the animals may have had pre-existing specific antibodies against these bacterial antigens, or the responses might involve cross-reactivity with similar pathogens, as suggested by the basal response in controls [38].

Elevated levels of IgG1 and IgG2a observed in vaccine groups, along with markers of Th1 (IFN-γ), Th2 (IL-5), and Th17 (IL-17) responses, suggest that the chimeric protein can elicit a mixed immune response to some extent enhanced by the use of the ISA50 adjuvant. This combination of humoral (IgG1 and IgG2a) and cellular (IFN-γ, IL-5, IL-17) responses indicates that a vaccine containing EspB and Int280γ—whether as recombinant antigens alone, fused, or anchored to the bacterial membrane—could provide broad and effective protection against the pathogen.

This study requires validation in cattle, the target species for the vaccine. However, the results are promising and provide a foundation for designing an optimized assay in cattle.

## 5. Conclusions

The membrane-anchoring chimera successfully generated specific antibodies against the fused and individual proteins of EHEC O157:H7, with no significant differences in immune response between the individual proteins. Expression of this chimera in *Salmonella* and ETEC enhances the potential of the NCD vaccine to protect calves against neonatal diarrhea. This approach could also benefit human health by helping prevent HUS without increasing production costs or altering the standard cattle vaccination schedule.

## Figures and Tables

**Table 1:** (**A**): Groups of vaccinated mice. (**B**): Groups of vaccinated guinea pigs.

(**A**)		
**Groups of Mice**	**Treatments**	**Details**
1	Control	150 µL of PBS
2	EspB and Int280γ	1 µg of EspB and 1 µg of Int280γ dissolved in 150 µL of PBS
3	Chimera protein (low dose)	2 µg of Chimera protein dissolved in 150 µL of PBS
4	Chimera protein (high dose)	10 µg of Chimera protein dissolved in 150 µL of PBS
5	Inactivated ETEC B41Arg expressing Chimera protein	1.10^8^ CFU of inactivated ETEC B41Arg expressing Chimera protein resuspended in PBS
6	Inactivated *Salmonella* Dublin expressing Chimera protein	1.10^8^ CFU of inactivated *Salmonella* Dublin expressing Chimera protein resuspended in PBS
7	Inactivated ETEC B41Arg and *Salmonella* Dublin expressing Chimera protein	1.10^8^ CFU of inactivated ETEC B41Arg and *Salmonella* Dublin, both expressing Chimera protein resuspended in PBS
8	Inactivated ETEC B41Arg and *Salmonella* Dublin expressing Chimera proteins + BRoVA UK and BCoVB Mebus	1.10^8^ CFU of inactivated ETEC B41Arg and *Salmonella* Dublin expressing Chimera proteins resuspended with 1.10^7^ FFU BRoVA UK and BCoVB Mebus
(**B**)		
**Groups of guinea pigs**	**Treatments**	**Details**
Control	Control	1 mL of PBS
Vaccinated	Inactivated ETEC B41Arg and *Salmonella* Dublin expressing Chimera proteins + BRoVA UK and BCoVB Mebus	1.10^8^ CFU of inactivated ETEC B41Arg and *Salmonella* Dublin expressing Chimera proteins resuspended with 1.10^7^ FFU BRoVA UK and BCoVB Mebus

## Data Availability

The original contributions presented in this study are included in the article/supplementary material. Further inquiries can be directed to the corresponding author(s).

## Supplementary Materials

The following supporting information can be downloaded at: https://www.mdpi.com/article/10.3390/vaccines13020124/s1, Figure S1 Nucleotide and translated sequences of chimeric protein, Figure S2, Detection of recombinant chimeric protein expression with specific EspB and Int280γserum from a bovine–Figure S3, Inactivation of recombinant ETEC, Figure S4 Antigenic preservation in vaccine formulation and S5: Specific IgG responses in inoculated mice
