# Supplementary material for: A Novel Vaccine for Bovine Diarrhea Complex Utilizing Recombinant Enterotoxigenic Escherichia coli and Salmonella Expressing Surface-Displayed Chimeric Antigens from Enterohemorrhagic Escherichia coli O157:H7
Source: Vaccines (Basel). 2025 Jan 25;13(2):124. doi: 10.3390/vaccines13020124 (PMC11860786; doi:10.3390/vaccines13020124)
Supplement: Supplementary file 1 [file vaccines-13-00124-s001.zip › Supplementary Figure S1.pdf]

1/**1** 31/**11** 61/**21** 91/**31**

GGATCCGGCCATGGCAGTGTCAAAAAAGTATTTGCCTTTGATTATCGCCTCAGTAGTTTTAACGGGGTGCACCATTCCGGGTTCACACCTACCTACAGGCGAAAAAAATGCCGCAATCGC

Wza

D P A M A V S K K Y L P L I I A S V V L T G C T I P G S H L P T G E K N A A I A 2>

121/**41** 151/**51** 181/**61** 211/**71**

ACTATTAGCATCTTTTGCTTTTGGTGGCGTAGCGATGGCTGCTGTTGAAGAGACAACAACAGCGTCAACCACTGGCGGAGCCGCTGGCGGCACAGCAGCGACAACGGCAGCAGTAGGTAC

Omp

L L A S F A F G G V A M A A V E E T T T A S T T G G A A G G T A A T T A A V G T 2>

241/**81** 271/**91** 301/**101** 331/**111**

AGTAACGGCACGGACTAGTCCGAATACTATTGATAATACTCAAGTAACGATGGTTAATTCCGCTTCGGAGAGTACGACCGGCGCTTCCAGTGCAGTTGCCGCATCTGCTTTATCAATTGA

EspB

V T A R T S P N T I D N T Q V T M V N S A S E S T T G A S S A V A A S A L S I D 2>

361/**121** 391/**131** 421/**141** 451/**151**

TTCATCTCTGCTTACTGATGGTAAGGTTGATATTTGTAAGCTGATGCTGGAAATTCAAAAACTCCTCGGCAAGATGGTGACTCTATTGCAGGATTACCAACAAAAACAATTGGCGCAAAG

S S L L T D G K V D I C K L M L E I Q K L L G K M V T L L Q D Y Q Q K Q L A Q S 2>

481/**161** 511/**171** 541/**181** 571/**191**

CTATCAGATTCAGCAGGCCGTTTTTGAGAGCCAGAATAAAGCTATTGAGGAAAAAAAAGCCGCGGCAACCGCTGCTTTGGTTGGCGGGATTATTTCATCAGCATTGGGGATCTTAGGTTC

EspB

Y Q I Q Q A V F E S Q N K A I E E K K A A A T A A L V G G I I S S A L G I L G S 2>

601/**201** 631/**211** 661/**221** 691/**231**

TTTTGCAGCAATGAACAACGCGGCTAAAGGGGCTGGTGAGATTGCTGAAAAAGCAAGCTCTGCATCTTCAAAGGCTGCTGGTGCGGCTTCTGAGGTTGCAAATAAAGCTCTGGTCAAGGC

F A A M N N A A K G A G E I A E K A S S A S S K A A G A A S E V A N K A L V K A 2>

721/**241** 751/**251** 781/**261** 811/**271**

TACGGAAAGTGTTGCTGATGTCGCAGAGGAGGCATCCAGTGCGATGCAGAAAGCGATGGCCACAACAACGAAAGCAGCCAGCCGTGCATCTGGCGTTGCAGATGATGTTGCGAAAGCCTC

EspB

T E S V A D V A E E A S S A M Q K A M A T T T K A A S R A S G V A D D V A K A S 2>

841/**281** | 871/**291** | 901/**301** | 931/**311**

TGACTTTGCTGAAGATCTTGCAGACGCCGCCGAGAAGACAAGCAGAATCAATAAGTTGTTGAATTCCGTAGATAAACTGACCAATACCACAGCATTTGTTGCCGTGACCAGTCTTGCTGA

D F A E D L A D A A E K T S R I N K L L N S V D K L T N T T A F V A V T S L A E 2>

961/**321** | 991/**331** | 1021/**341** | 1051/**351**

AGGTACGAAAACGTTGCCAACAACAATATCTGAGTCCGTCAAATCGACTCATGAGGTTAATGAACAACGTGCGAAGTCGCTGGAAAACTTCCAGCAGGGGAATCTGGAGCTGTATAAACA

EspB

G T K T L P T T I S E S V K S T H E V N E Q R A K S L E N F Q Q G N L E L Y K Q 2>

1081/**361** | 1111/**371** | 1141/**381** | 1171/**391**

AGACGTTCGCAGAACGCAGGATGATATCACGACTCGTCTGCGTGATATAACGTCCGCTGTCCGCGATCTCCTTGAGGTCCAGAATCGTATGGGGCAATCGGGTCGCTTAGCTGGGGAAGC

D V R R T Q D D I T T R L R D I T S A V R D L L E V Q N R M G Q S G R L A G E A 2>

1201/**401** | 1231/**411** | 1261/**421** | 1291/**431**

CGCCGCCAAAGAAGCCGCCGCCAAAGAAGCCGCCGCCAAAGAAGCCGCCGCCAAACAAACCAAGGCCAGCATTACTGAGATTAAGGCTGATAAGACAACTGCAGTAGCAAATGGTAAGGA

linker

A A K E A A A K E A A A K E A A A K Q T K A S I T E I K A D K T T A V A N G K D 2>

1321/**441** | 1351/**451** | 1381/**461** | 1411/**471**

TGCTATTAAATATACTGTAAAAGTTATGAAAAACGGTCAGCCAGTTAATAATCAATCCGTTACATTCTCAACAAACTTTGGGATGTTCAACGGTAAGTCTCAAACGCAAGCAACCACGGG

Int 280

A I K Y T V K V M K N G Q P V N N Q S V T F S T N F G M F N G K S Q T Q A T T G 2>

1441/**481** | 1471/**491** | 1501/**501** | 1531/**511**

AAATGATGGTCGTGCGACGATAACACTAACTTCCAGTTCCGCCGGTAAAGCGACTGTTAGTGCGACAGTCAGTGATGGGGCTGAGGTTAAAGCGACTGAGGTCACTTTTTTTGATGAACT

N D G R A T I T L T S S S A G K A T V S A T V S D G A E V K A T E V T F F D E L 2>

1561/**521** | 1591/**531** | 1621/**541** | 1651/**551**

GAAAATTGACAACAAGGTTGATATTATTGGTAACAATGTCAGAGGCGAGTTGCCTAATATTTGGCTGCAATATGGTCAGTTTAAACTGAAAGCAAGCGGTGGTGATGGTACATATTCATG

Int 280

K I D N K V D I I G N N V R G E L P N I W L Q Y G Q F K L K A S G G D G T Y S W 2>

1681/**561** | 1711/**571** | 1741/**581** | 1771/**591**

GTATTCAGAAAATACCAGTATCGCGACTGTCGATGCATCAGGGAAAGTCACTTTGAATGGTAAAGGCAGTGTCGTAATTAAAGCCACATCTGGTGATAAGCAAACAGTAAGTTACACTAT

```
 Y  S  E  N  T  S  I  A  T  V  D  A  S  G  K  V  T  L  N  G  K  G  S  V  V  I  K  A  T  S  G  D  K  Q  T  V  S  Y  T  I   2>

1801/601                      1831/611                      1861/621                      1891/631
|                             |                             |                             |
AAAAGCACCGTCGTATATGATAAAAGTGGATAAGCAAGCCTATTATGCTGATGCTATGTCCATTTGCAAAAATTTATTACCATCCACACAGACGGTATTGTCAGATATTTATGACTCATG
                                                                  Int 280
 K  A  P  S  Y  M  I  K  V  D  K  Q  A  Y  Y  A  D  A  M  S  I  C  K  N  L  L  P  S  T  Q  T  V  L  S  D  I  Y  D  S  W   2>

1921/641                      1951/651                      1981/661                      2011/671
|                             |                             |                             |
GGGGGCTGCAAATAAATATAGCCATTATAGTTCTATGAACTCAATAACTGCTTGGATTAAACAGACATCTAGTGAGCAGCGTTCTGGAGTATCAAGCACTTATAACCTAATAACACAAAA
 G  A  A  N  K  Y  S  H  Y  S  S  M  N  S  I  T  A  W  I  K  Q  T  S  S  E  Q  R  S  G  V  S  S  T  Y  N  L  I  T  Q  N   2>

2041/681                      2071/691                      2101/701
|                             |                             |
CCCTCTTCCTGGGGTTAATGTTAATACTCCAAATGTCTATGCGGTTTGTGTAGAAGCTCTAGAGAAGCTT
                    Int 280
 P  L  P  G  V  N  V  N  T  P  N  V  Y  A  V  C  V  E  A  L  E  K  L   2>
```

Wza nt 11- 82
Omp nt 83- 217
EspB nt 218 - 1195
Linker nt 1196 -1258
Int 280 1259 -2095
