# Supplementary material for: A Novel Vaccine for Bovine Diarrhea Complex Utilizing Recombinant Enterotoxigenic Escherichia coli and Salmonella Expressing Surface-Displayed Chimeric Antigens from Enterohemorrhagic Escherichia coli O157:H7
Source: Vaccines (Basel). 2025 Jan 25;13(2):124. doi: 10.3390/vaccines13020124 (PMC11860786; doi:10.3390/vaccines13020124)
Supplement: Supplementary file 1 [file vaccines-13-00124-s001.zip › Supplementary Figure S2.pdf]

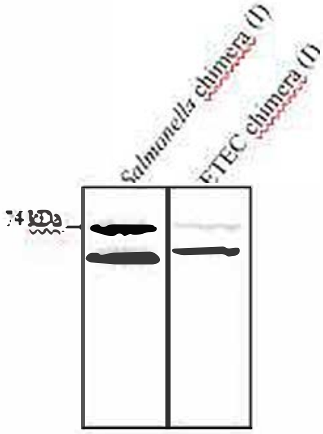

Supplementary figure S2: Detection of recombinant chimeric protein expression with specific EspB and Int280γ serum from a bovine. ETEC and *Salmonella* transformed with pTrcHis2B-BLI280 were induced (I) with IPTG to express the recombinant chimera. The samples were separated by SDS-PAGE. Chimera detection was performed using a positive EspB and Int280γ serum, as primary antibody, and an alkaline phosphatase-conjugated anti-bovine, as secondary antibody, in all cases.
