# Supplementary material for: A Novel Vaccine for Bovine Diarrhea Complex Utilizing Recombinant Enterotoxigenic Escherichia coli and Salmonella Expressing Surface-Displayed Chimeric Antigens from Enterohemorrhagic Escherichia coli O157:H7
Source: Vaccines (Basel). 2025 Jan 25;13(2):124. doi: 10.3390/vaccines13020124 (PMC11860786; doi:10.3390/vaccines13020124)
Supplement: Supplementary file 1 [file vaccines-13-00124-s001.zip › Supplementary Figure S4.pdf]

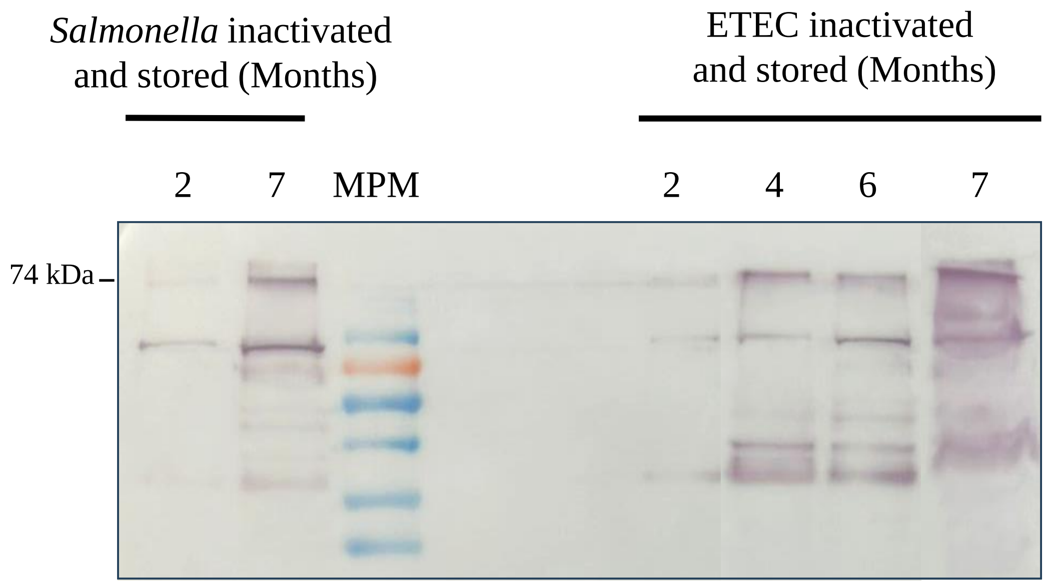


**Supplementary figure S4: Antigenic preservation in vaccine formulation.** ETEC and *Salmonella* transformed with pTrcHis2B-BLI280 were induced with IPTG to express the recombinant chimera. The samples were inactivated, stored at 4 ºC and separated by SDS-PAGE. Chimera detection was performed using a mouse-specific anti Hisx6-tag primary antibody and an alkaline phosphatase-conjugated anti-mouse as a secondary antibody.
