# Supplementary material for: A Novel Vaccine for Bovine Diarrhea Complex Utilizing Recombinant Enterotoxigenic Escherichia coli and Salmonella Expressing Surface-Displayed Chimeric Antigens from Enterohemorrhagic Escherichia coli O157:H7
Source: Vaccines (Basel). 2025 Jan 25;13(2):124. doi: 10.3390/vaccines13020124 (PMC11860786; doi:10.3390/vaccines13020124)
Supplement: Supplementary file 1 [file vaccines-13-00124-s001.zip › Supplementary Figure S5.pdf]

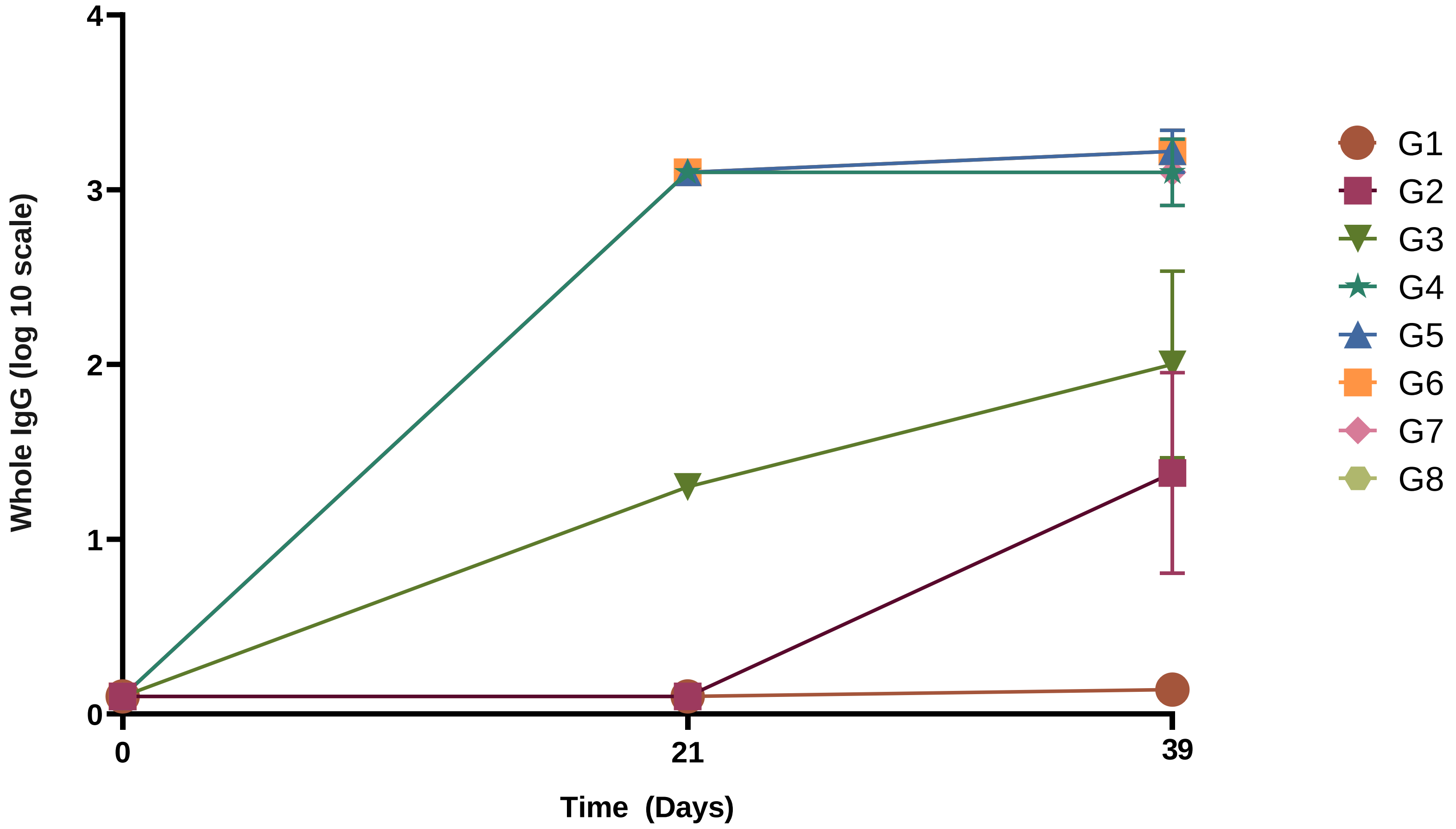

Supplementary figure S5: Specific IgG responses in inoculated mice. ELISA plates were coated with purified the recombinant chimera. The chimera-specific antibody response was measured for each group by indirect ELISA of a pool of serum samples from 21 and 39 dpv mice. Group 1: 150 µl of PBS (Control), Group 2: 1 µg of EspB and 1 µg of Int280γ, Group 3: 2 µg of the chimera, Group 4: 10 µg of the chimera, Group 5: 1.10^8 inactivated CFU of ETEC B41 expressing the chimera, Group 6: 1.10^8 inactivated CFU of *Salmonella* dublin expressing the chimera, Group 7: 1.10^8 inactivated CFU of ETEC B41 and *Salmonella* dublin, both expressing the chimera and Group 8: 1.10^8 inactivated CFU of ETEC B41 and *Salmonella* dublin, both expressing the chimera, plus 1.10^7 FFU BRoVA UK and BCoVB Mebus. Goat anti-mouse IgG conjugated with horseradish peroxidase was used as a secondary antibody. ABTS was used as substrate and the reaction was measured at OD450. The antibody titer was expressed as the reciprocal of the end-point dilution resulting in an OD405.
